# RANBP3 and RAN orchestrate CRM1-mediated nuclear export of hepatitis B virus RNAs

**DOI:** 10.1128/jvi.00267-26

**Published:** 2026-04-30

**Authors:** Mengfei Wang, Quan Chen, Yingcheng Zheng, Jiatong Yin, Guoguo Zhu, Kaitao Zhao, Zaichao Xu, Rong Hua, Meng Zhou, Lu Zhang, Gaihong Zhao, Shipeng Yang, Zulihuma Amat, Yuchen Xia, Xiaoming Cheng

**Affiliations:** 1 State Key Laboratory of Virology and Biosafety, Hubei Provincial Research Center for Basic Biological Sciences and Hubei Province Key Laboratory of Allergy and Immunology, Institute of Medical Virology, TaiKang Center for Life and Medical Sciences, TaiKang Medical School, Wuhan University, Wuhan, China; 2 School of Life Sciences, Hubei University 117923, Wuhan, China; 3 Department of Emergency, General Hospital of Central Theater Command of People’s Liberation Army of China, Wuhan, China; 4 Pingyuan Laboratory, Henan, China; 5 Wuhan University Center for Pathology and Molecular Diagnostics, Zhongnan Hospital of Wuhan University 89674, Wuhan, China; Wake Forest University School of Medicine, Winston-Salem, North Carolina, USA

**Keywords:** HBV RNA, RANBP3-RAN-CRM1 pathway, RNA export

## Abstract

Nuclear export of viral RNAs is essential for the replication of hepatitis B virus (HBV). Our previous study demonstrated that ELAVL1 (embryonic lethal, abnormal vision, Drosophila-like 1) mediates the nuclear export of HBV RNAs via the chromosome region maintenance 1 (CRM1) pathway by recognizing and binding to the AUUUA motifs within these transcripts. Here, we identify Ras-related nuclear protein (RAN) and RAN-binding protein 3 (RANBP3) as critical downstream regulators of this CRM1-mediated RNA export pathway. We show that RANBP3 recruits RAN-GTP to assemble an export-competent quaternary complex (CRM1-HBV RNAs-RANBP3-RAN-GTP). The functional significance of this complex is underscored by the findings that its disruption, through knockdown of RANBP3/RAN, or by introducing CRM1 mutations that prevent cofactor binding, severely impairs HBV RNA export and viral replication. Together, our study thus elucidates a precise regulatory mechanism governing HBV RNA trafficking and highlights RANBP3 and RAN as potential antiviral targets.

**IMPORTANCE:**

Efficient nuclear export of hepatitis B virus (HBV) RNAs is essential for viral replication, yet the regulatory mechanisms controlling this process remain poorly defined. This study identifies Ras-related nuclear protein (RAN) and RAN-binding protein 3 (RANBP3) as key host cofactors that drive chromosome region maintenance 1 (CRM1)-mediated export of HBV transcripts by assembling an export-competent complex with viral RNAs. Disrupting this pathway profoundly impairs RNA export and downstream steps of the viral life cycle. By defining how HBV harnesses the RANBP3-RAN-CRM1 axis for RNA trafficking, our work reveals a previously unrecognized layer of host dependency and highlights RANBP3 and RAN as promising targets for antiviral intervention.

## INTRODUCTION

Hepatitis B virus (HBV) infection is a global public health issue and a major cause of hepatocellular carcinoma and cirrhosis (1). At present, the principal therapeutic drugs for HBV infection, including nucleos(t)ide analogs (NAs) and pegylated interferon-α (peg-IFN-α), are incapable of achieving the clearance of viral infection (2, 3).

HBV is classified within the Hepadnaviridae family and is characterized as a small, enveloped DNA virus (4). The genome of the virus is a partially double-stranded DNA of 3.2 kb, containing four overlapping open reading frames (ORFs): preS1/preS2/S, preC/C, P, and X, which encode the viral surface protein, core protein, polymerase, and X protein, respectively. Furthermore, the preC ORF also encodes the pre-core protein, which is subsequently processed into the hepatitis B e antigen (HBeAg) (1, 5). The life cycle of HBV initiates with specific binding of the viral receptor, sodium-taurocholate cotransporting polypeptide (NTCP), on the hepatocyte surface and enters into the cell through clathrin-mediated endocytosis (6). Subsequently, HBV releases the nucleocapsid and transports its genome into the nucleus. In here, HBV partially double-stranded relaxed circular DNA (rcDNA) is repaired by host proteins to form covalently closed circular DNA (cccDNA) (7, 8). HBV cccDNA serves as the transcriptional template to produce viral RNAs, including pregenomic RNA (pgRNA), precore RNA, L/M/S mRNAs, and X mRNA (9). These RNAs are then transported to the cytoplasm and translated into viral proteins. In the cytoplasm, pgRNA and polymerase are encapsulated by the core protein to form new nucleocapsids. The viral polymerase employs pgRNA as a template for reverse transcription to synthesize rcDNA (10). The newly synthesized nucleocapsids are enveloped by surface proteins in the multivesicular body and secreted as progeny virus particles or return to the nucleus to replenish the cccDNA pool (5, 11).

The nuclear export of HBV RNAs is a crucial step that enables the virus to synthesize its proteins through cytoplasmic translation and replicate its genome via reverse transcription. Despite its importance, the molecular mechanisms orchestrating HBV RNA export remain incompletely understood. In our previous work, we employed an unbiased quantitative proteomics approach and identified the host RNA-binding protein ELAVL1 as a key factor that recognizes HBV transcripts containing AUUUA motifs. Through this interaction, ELAVL1 stabilizes viral RNAs and facilitates their nuclear export via the CRM1-dependent pathway (12). Nevertheless, the downstream regulatory machinery that provides directionality and energy for this nucleocytoplasmic transport process has not been clearly defined. In particular, how CRM1 cooperates with other essential components of the nuclear export system, which drives export complex assembly and disassembly, remains to be elucidated in the context of HBV infection.

In this study, we revisited our HBV pgRNA-interaction proteomics data and identified Ran-binding protein 3 (RANBP3) as a potential factor involved in HBV RNA nuclear export. Mechanistically, RANBP3 recruits RAN-GTP to assemble a stable export-competent complex composed of CRM1-HBV RNAs-RANBP3-RAN-GTP, which mediates efficient RNA translocation through the nuclear pore complex. Disruption of any component, via RANBP3/RAN depletion or CRM1 mutations that block cofactor binding, leads to defective HBV RNA export, diminished viral antigen release, and reduced viral DNA replication. This mechanism defines a coordinated ELAVL1-RANBP3-RAN-CRM1 axis that governs HBV RNA trafficking and identifies RANBP3 and RAN as potential antiviral targets.

## RESULTS

### RANBP3 is a host factor regulating the nuclear export of HBV RNAs

**Fig 1:**
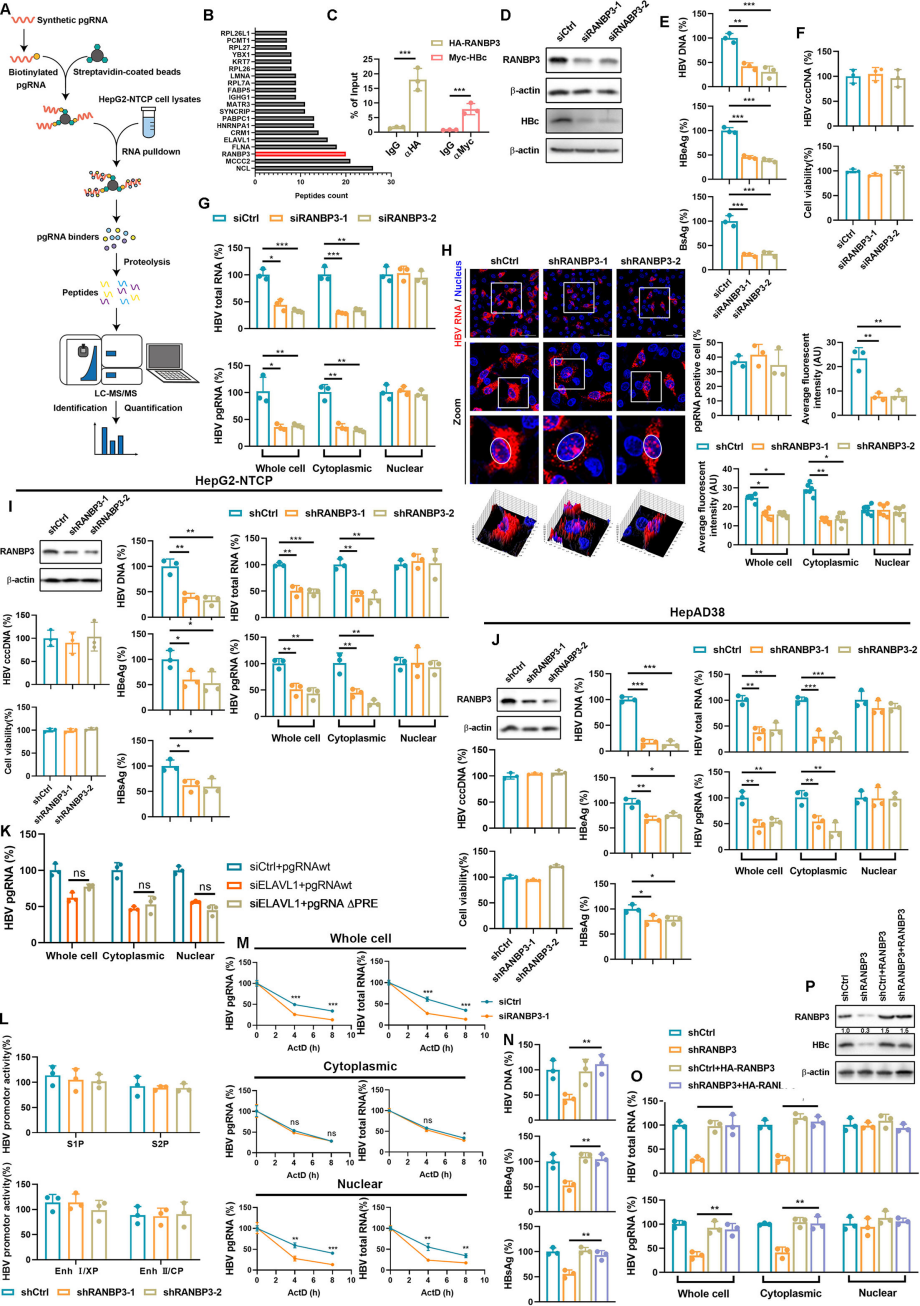
Knock down of RANBP3 reduces the nuclear export of HBV RNAs. (**A**) Diagram illustrating pgRNA pull-down LC-MS/MS. (**B**) The number of pgRNA-binding peptides identified by RNA-pulldown-LC-MS/MS. (**C**) Huh7 cells were co-transfected with HA-RANBP3 and Myc-HBc with the pCDNA3.1-pgRNA plasmid. Use RIP to detect the interaction between RANBP3-pgRNA and HBc-pgRNA. (**D–G**) HepG2-NTCP cells were transfected with siRANBP3 for 2 days and then infected with HBV at an MOI of 200. The cells were maintained in 2.5% DMSO DMEM for 5 days. (**D**) RANBP3 and HBc protein levels were detected by WB. (**E**) HBV DNA level in the supernatant was detected by qPCR, and the levels of HBeAg and HBsAg in supernatant were detected by ELISA. (**F**) The level of HBV cccDNA was detected by qPCR. The cell viability was detected by CCK-8 assay. (**G**) HBV RNAs in cytoplasm and nucleus were detected by qPCR. (**H**) HepG2-NTCP cells were transduced with lentivirus-shCtrl or lentivirus-shRANBP3 and infected with HBV at MOI = 200. RNAscope assay was used to detect the subcellular distribution of HBV RNAs. Nuclei were stained with Hoechst 33258. Data were shown as mean ± SEM. *: *P* < 0.05; **: *P* < 0.01. (**I**) HepG2-NTCP cells were transduced with lentivirus-shRANBP3 and infected with HBV. The replication markers of HBV were detected. (**J**) HepAD38 cells were transduced with lentivirus-shRANBP3 for 5 days, and the replication markers of HBV were determined. (**K**) Huh7 cells were transfected with pCDNA3.1-pgRNA wild-type plasmid or pCDNA3.1-pgRNA delete PRE plasmid upon ELAVL1 knockdown. HBV pgRNA in the cytoplasm and nucleus was detected by qPCR. (**L**) Huh7 cells were transfected with shCtrl or shRANBP3 for 12 h and then transfected with HBV promoter/enhancer vectors for 24 h. Firefly luciferase activity was measured. (**M**) HepAD38 cells were transfected with siRANBP3 for 5 days and then treated with 10 nM actinomycin D for the indicated time points. qPCR assay was used to detect the level of HBV RNAs in the cytoplasm and nucleus. (**N–P**) RANBP3 stable knockdown HepG2-NTCP cells were transduced with lentivirus-HA-RANBP3 pretreated with 2.5% DMSO for 2 days and then infected HBV at MOI = 200. The cells were maintained in DMEM containing 2.5% DMSO for 5 days. (**N**) The level of HBV DNA in supernatant was detected by qPCR, and the levels of HBeAg and HBsAg in the supernatant were detected by ELISA. (**O**) The level of HBV RNAs in cell fractions was detected by qPCR. (**P**) The levels of RANBP3 and HBc were detected by WB. Data were shown as mean ± SD. *: *P* < 0.05, **: *P* < 0.01, and ***: *P* < 0.001.

We previously identified ELAVL1 as a host factor that binds AUUUA-containing HBV transcripts, stabilizing them and promoting their CRM1-dependent nuclear export (12). To further define the machinery that provides directionality and coordinates CRM1 with other nuclear export components, we analyzed our pgRNA interactome (Fig. 1A). RANBP3 emerged as one of the most enriched factors associated with nuclear RNA export (Fig. 1B). A robust interaction between RANBP3 and HBV pgRNA was confirmed by RNA immunoprecipitation (RIP) (Fig. 1C). Knockdown of RANBP3 markedly decreased production of HBc (Fig. 1D), as well as of HBeAg, HBsAg, and secreted HBV DNA in HBV-infected HepG2-NTCP cells (Fig. 1E) without affecting HBV cccDNA and cell viability (Fig. 1F). Cytoplasmic-nuclear fractionation demonstrated a substantial decrease in cytoplasmic HBV RNAs without changes in nuclear RNA levels (Fig. 1G), which was further supported by RNAscope analysis (Fig. 1H). Similar results were obtained following shRNA-mediated depletion of RANBP3 in HBV-infected HepG2-NTCP cells (Fig. 1I) and in HepAD38 (Fig. 1J), an HBV-expressing cell line. As PRE is a well-known HBV RNA nuclear export regulatory element (13–17), to determine whether PRE cooperates with the ELAVL1-CRM1 pathway in regulating HBV RNA export, we generated a pCDNA3.1-pgRNA construct lacking the PRE sequence. Consistent with prior reports, deletion of the PRE alone markedly impaired pgRNA export. However, upon PRE deletion, knockdown of ELAVL1 did not further impair pgRNA nuclear export. This finding suggests that the PRE-mediated export pathway functions independently of the ELAVL1-CRM1 axis (Fig. 1K). RANBP3 knockdown did not affect HBV promoter/enhancer activity (Fig. 1L) but expedited the degradation of HBV RNAs in the whole cell and nucleus but not in the cytoplasm when treated with the transcription inhibitor actinomycin D (Fig. 1M). Importantly, re-expression of RANBP3 restored HBV RNA nuclear export and rescued viral replication (Fig. 1N through P). Collectively, these findings demonstrate that RANBP3 governs the nuclear export of HBV RNAs and is essential for efficient HBV replication.

### Ranbp3 regulates HBV RNA *in vivo*

**Fig 2:**
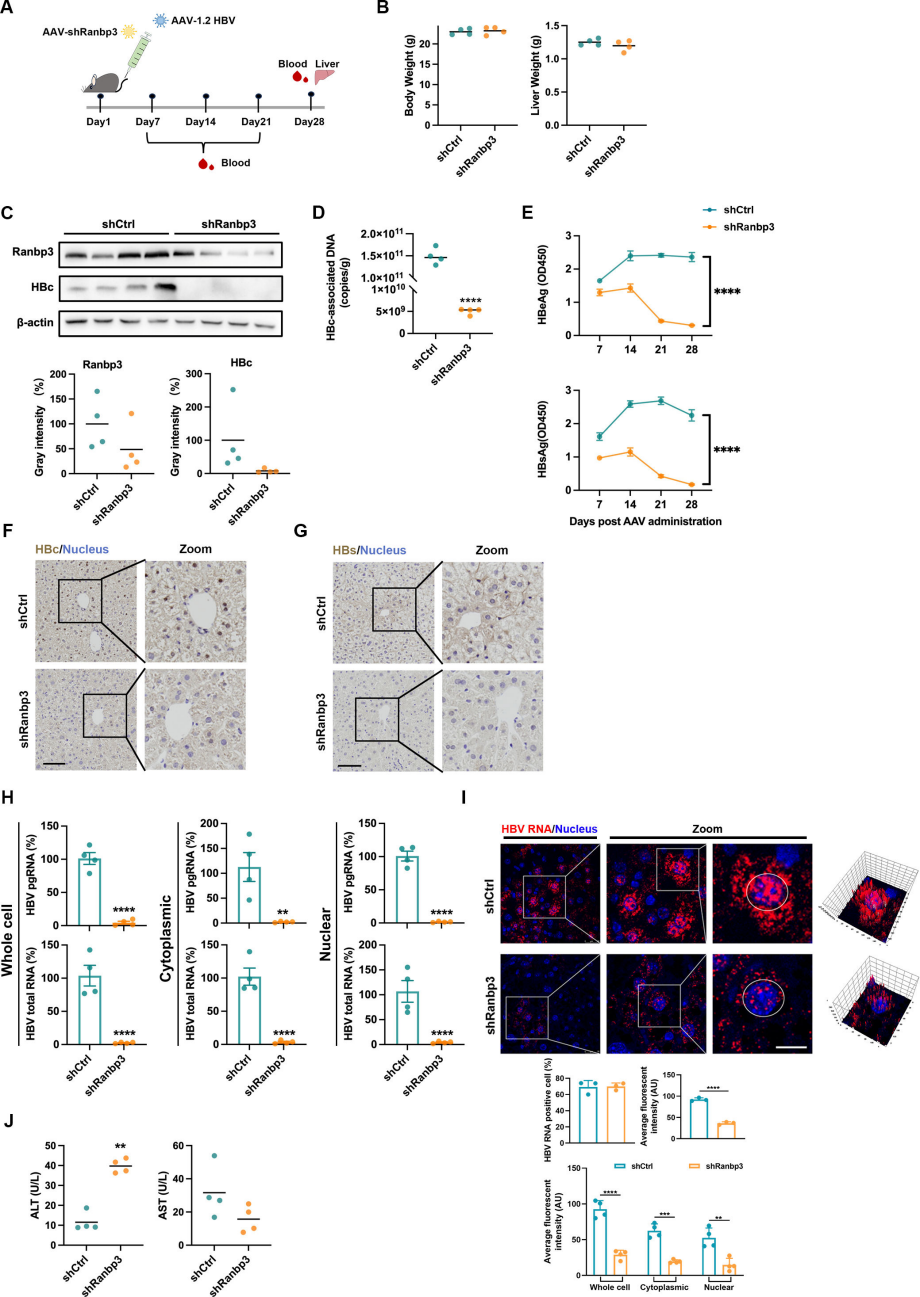
Ranbp3 regulates HBV RNA nuclear export *in vivo*. (**A**) Schematic diagram of the mouse experiment. AAV-shCtrl or AAV-shRanbp3 was co-transduced with AAV-1.2HBV into C57BL/6J mice (*n* = 4). Blood samples were collected from the orbital veins once a week, and the mice were sacrificed in the fourth week. (**B**) Body weight and liver weight of mice. (**C**) The levels of intrahepatic Ranbp3 and HBc were detected by WB. (**D**) HBc-associated DNA in intrahepatic samples was determined by qPCR. (**E**) HBeAg (50× dilution) and HBsAg (2,000× dilution) in serum were detected by ELISA. (**F and G**) Liver was collected and fixed, stained with HBc antibody or HBs antibody, and examined by light microscopy. (**H**) Intrahepatic level of HBV RNAs in the cytoplasmic and nucleus was detected by qPCR. (**I**) RNAscope assay was used to detect the intrahepatic distribution of HBV RNA. Nuclei were stained with Hoechst 33258. (**J**) Levels of ALT and AST in serum were determined by ELISA. Data were shown as mean ± SEM. *: *P* < 0.05, **: *P* < 0.01, ***: *P* < 0.001, and ****: *P* < 0.0001.

To further assess the role of RANBP3 *in vivo*, we employed a mouse model in which AAV-HBV1.2 and AAV-shRanbp3 were delivered via tail-vein injection (Fig. 2A). Ranbp3 knockdown did not affect body weight or liver weight (Fig. 2B) but led to a substantial reduction in both Ranbp3 and HBc protein levels (Fig. 2C). Consistently, liver HBc-associated DNA levels were markedly suppressed (Fig. 2D), and enzyme-linked immunosorbent assay (ELISA) revealed a significant reduction in serum HBeAg and HBsAg (Fig. 2E). Immunohistochemistry further confirmed diminished HBc (Fig. 2F) and HBs (Fig. 2G) signals in liver tissue. However, hepatocyte nuclear-cytoplasmic fractionation (Fig. 2H) and RNAscope assays (Fig. 2I) showed significant reductions in both cytoplasmic and nuclear HBV RNAs, differing from our *in vitro* observations, and were accompanied by elevated ALT levels (Fig. 2J), indicating liver injury. Despite these differences, the overall data support a role for Ranbp3 in regulating HBV RNA nuclear export *in vivo*.

### HBV manipulates the RAN-dependent nucleocytoplasmic transport system for replication

**Fig 3:**
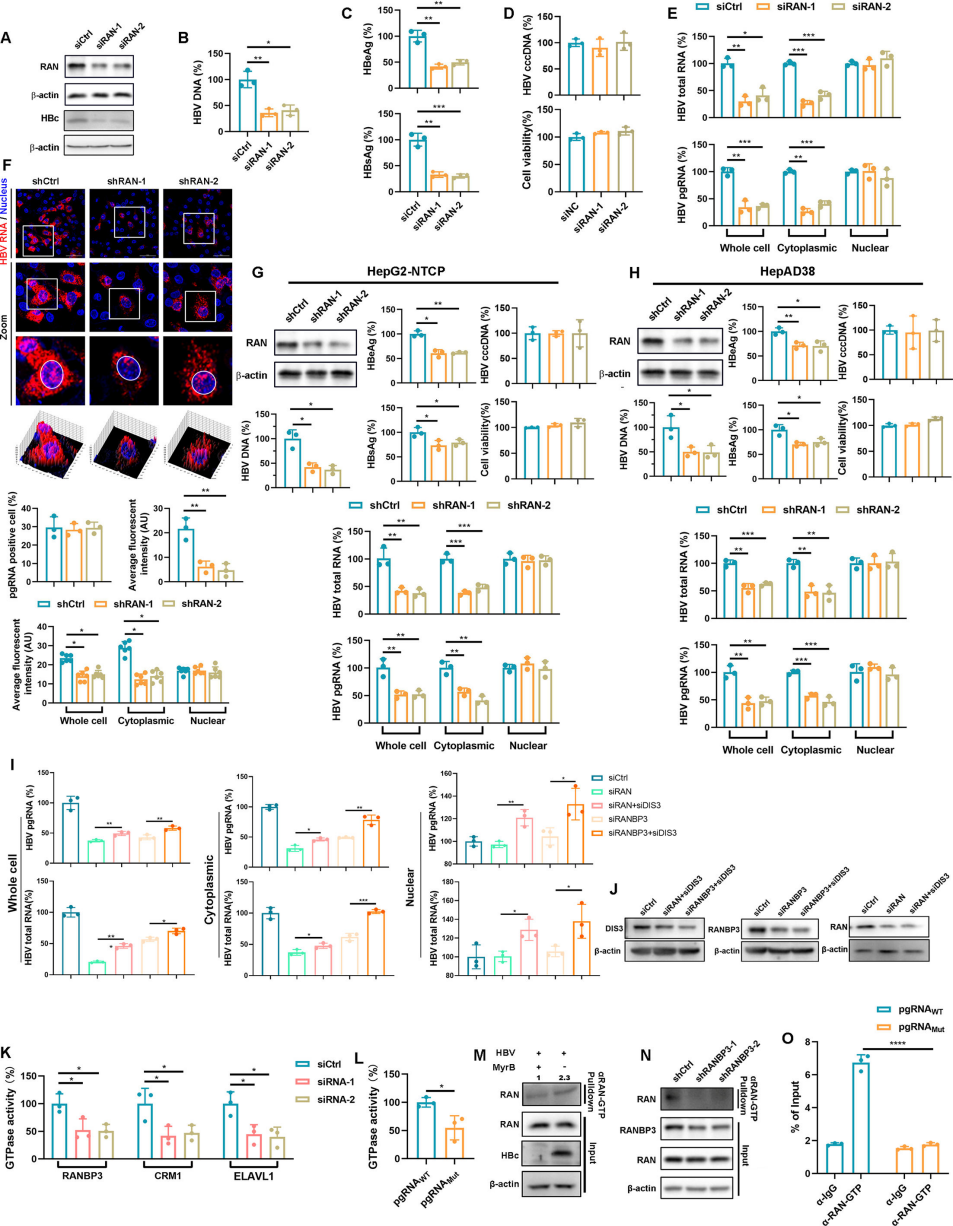
HBV triggers RAN activation to facilitate its replication. (**A–D**) HepG2-NTCP cells were transfected with siRNA for a period of 2 days, following which the cells were infected with HBV at an MOI of 200. The cells were cultivated in 2.5% DMSO DMEM for 5 days. (**A**) WB was used to detect the protein level of RAN and HBc. (**B**) The HBV DNA levels in the supernatant were detected by qPCR. (**C**) The HBeAg and HBsAg levels in the supernatant were detected by ELISA. (**D**) The level of HBV cccDNA was detected by qPCR. The cell viability was detected by CCK-8 assay. (**E**) HBV RNA levels in cell fractions were detected by qPCR. (**F**) HepG2-NTCP cells were transduced with lentivirus-shCtrl or lentivirus-shRAN and infected with HBV. RNAscope assay was used to detect HBV RNAs in different cell fractions. Nuclei were stained with Hoechst 33258. Data were shown as mean ± SEM. *: *P* < 0.05; **: *P* < 0.01. (**G and H**) HBV replication markers were detected. (**G**) HepG2-NTCP cells were transduced with lentivirus-shRAN and infected with HBV. (**H**) HepAD38 cells were transduced with lentivirus-shRAN. (**I and J**) HepG2-NTCP cells were transfected with RAN or RANBP3 target siRNA upon DIS3 knockdown. The cells were infected with HBV for 5 days. (**I**) HBV RNA levels in cell fractions were detected by qPCR. (**J**) The protein levels of RAN, RANBP3, and DIS3 were detected by WB. (**J and K**) GTPase Activity Assay Kit (Colorimetric) was used to detect the cell’s GTPase activity. (**K**) HepAD38 cells were transfected with RANBP3, CRM1, and ELAVL1 targeted siRNAs. Then, the cells were maintained with DMEM supplemented with 2.5% DMSO for 5 days. (**L**) Huh7 cells were transfected with pgRNA WT or pgRNA MUT for 2 days. (**M and N**) RAN-GTP antibody was used to pulldown RAN-GTP. (**M**) HepG2-NTCP cells were infected with HBV at an MOI of 200 treated with Myrcludex B or not and were maintained with DMEM supplemented with 2.5% DMSO for 5 days. (**N**) HepG2-NTCP cells were transfected with siRANBP3 and infected with HBV at an MOI of 200 and were maintained with DMEM supplemented with 2.5% DMSO for 5 days. (**O**) Huh7 cells were transfected with pCDNA3.1-pgRNA WT or pCDNA3.1-pgRNA MUT plasmid for 2 days. RIP-qPCR assay was used to detect the interaction between RAN-GTP and pgRNA WT or pgRNA MUT. Data were shown as mean ± SD. *: *P* < 0.05, **: *P* < 0.01, ***: *P* < 0.001, and ****: *P* < 0.0001.

RANBP3 functions as a RAN-GTP binding protein that enhances the assembly and stability of nuclear export complexes, whereas RAN is a small GTPase that confers directionality to nucleocytoplasmic transport, including the CRM1 pathway (18, 19). To determine whether RAN participates in HBV RNA export, we knocked down RAN in HBV-infected HepG2-NTCP cells (Fig. 3A). RAN depletion impaired HBV replication, markedly reducing HBc expression (Fig. 3A), decreasing HBV DNA (Fig. 3B), as well as HBeAg and HBsAg secretion (Fig. 3C), without affecting HBV cccDNA and cell viability (Fig. 3D). Nuclear-cytoplasmic fractionation revealed a pronounced reduction in cytoplasmic HBV RNAs, while nuclear RNA levels remained unchanged (Fig. 3E), a finding further validated by RNAscope analysis (Fig. 3F). Similar results were obtained in HBV-infected HepG2-NTCP cells (Fig. 3G) and HepAD38 cells (Fig. 3H) with RAN-targeted lentivirus-shRNA transduced. In our previous study, we demonstrated that blockade of HBV RNA nuclear export does not result in nuclear accumulation because the retained transcripts are rapidly degraded by the nuclear RNA exosome complex, particularly through the DIS3 and RRP6 exonucleases (12). We further knocked down RAN or RANBP3 in the DIS3 knockdown cells to verify this conclusion (Fig. 3I and J). To assess whether HBV RNA export influences RAN GTPase activation, we measured intracellular GTPase activity. Knockdown of RANBP3, CRM1, and ELAVL1, the key factors involved in HBV RNA nuclear export, significantly reduced GTPase activity in HepAD38 cells (Fig. 3K). Moreover, pgRNA-WT exhibited higher intracellular GTPase activity compared to pgRNA-MUT (bearing mutations in the ELAVL1 recognition motif AUUUA) (Fig. 3L). Using a conformation-specific monoclonal antibody against RAN-GTP, we found that HBV infection activated RAN (Fig. 3M), whereas RANBP3 knockdown diminished the active RAN pool (Fig. 3N). Finally, the RIP assay demonstrated an interaction between RAN-GTP and pgRNA-WT, but not pgRNA-MUT (Fig. 3O). Together, these results indicate that HBV activates RAN and that RAN in turn facilitates efficient HBV RNA export and viral replication.

### Activated RAN is essential for HBV RNA nuclear export

**Fig 4:**
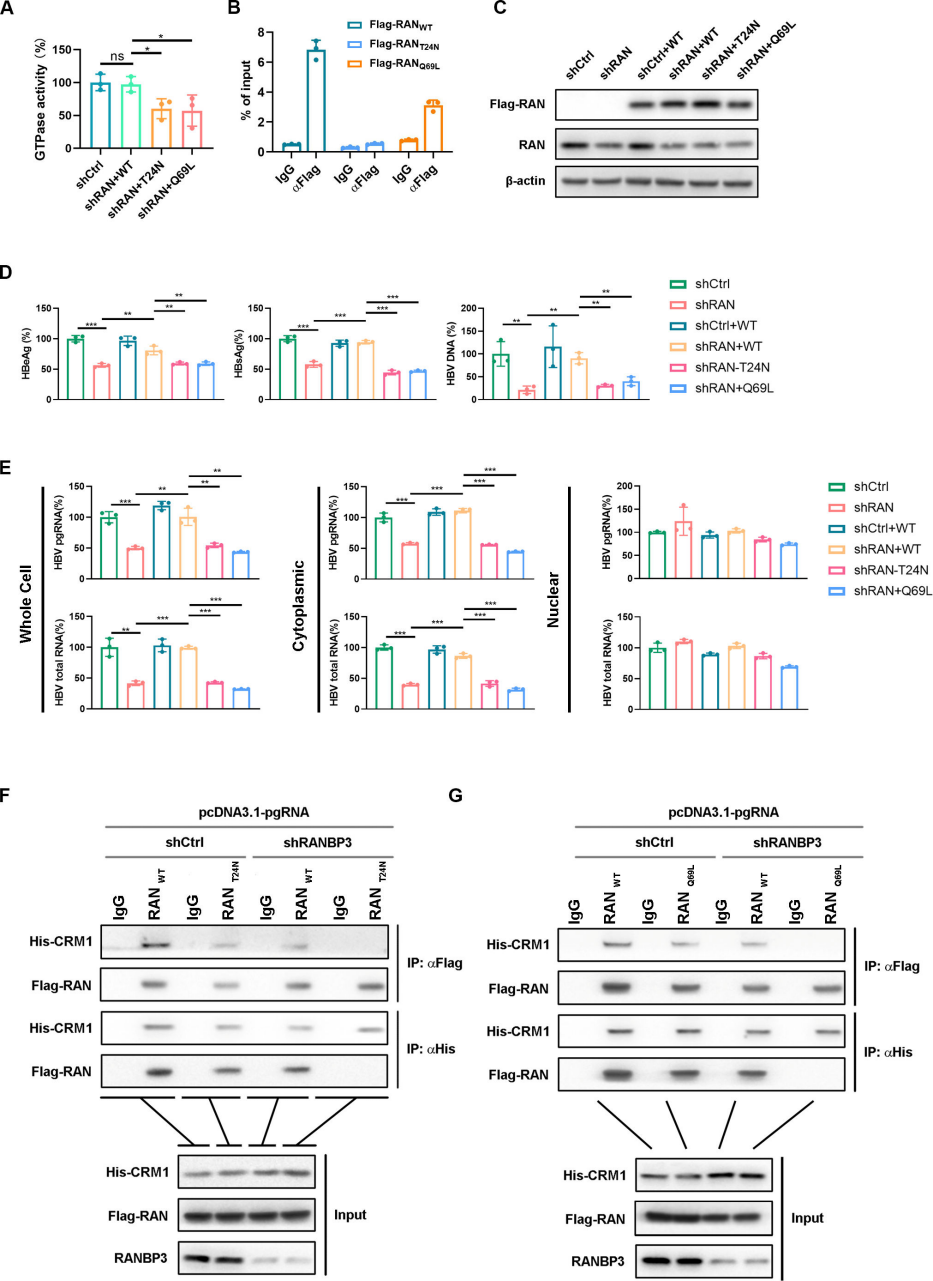
The activated RAN participates in the nuclear export of HBV RNAs. (**A**) Huh7 cells were transfected with shRAN for 12 h and then transfected with RAN WT or RAN MUT for 48 h. The GTPase Activity Assay Kit (Colorimetric) was used to detect the cell’s GTPase activity. (**B**) Huh7 cells were co-transfected with Flag-RAN, Flag-RAN T24N, and Flag-RAN Q69L with pCDNA3.1-pgRNA for 48 h. Then, the RIP assay was employed to detect the interaction between RAN and pgRNA. (**C–E**) Huh7 cells were transfected with shRAN for 12 h and then transfected with Flag-RAN WT, Flag-RAN T24N, and Flag-RAN Q69L with HBV 1.2 for 6 days. (**C**) RAN and Flag-RAN protein levels were detected by WB. (**D**) Levels of HBV DNA in the supernatant were detected by qPCR. Secreted HBeAg and HBsAg were determined by ELISA. (**E**) The level of HBV RNA in the cell fraction was detected by qPCR. (**F**) RANBP3 knockdown Huh7 cells were co-transfected with either Flag-RAN WT or Flag-RAN T24N with His-CRM1 and pCDNA3.1-pgRNA for 48 h. Co-IP assay was used to determine the interaction between RAN and CRM1. (**G**) RANBP3 knockdown Huh7 cells were co-transfected either Flag-RAN WT or Flag-RAN Q69L with His-CRM1 and pCDNA3.1-pgRNA for 48 h. Co-IP assay was used to determine the interaction between RAN and CRM1. Data were shown as mean ± SD.*: *P* < 0.05, **: *P* < 0.01, and ***: *P* < 0.001.

To confirm the involvement of RAN-GTP in the nuclear export of HBV RNAs, we constructed two RAN mutants: RAN Q69L, a GTP hydrolysis-defective mutant with extremely low GTPase activity (20), and RAN T24N, a nucleotide exchange defective mutant that mainly exists in the GDP-bound form (21, 22). The GTPase activity assay confirmed the successful generation of the two mutants (Fig. 4A). RIP assay demonstrated that, compared with the RAN WT, both mutants exhibited a substantial reduction in their capacity to bind to pgRNA (Fig. 4B). Furthermore, knockdown of RAN followed by supplementation with either mutant (Fig. 4C) fails to rescue HBV DNA and antigens levels in the supernatant (Fig. 4D), as well as the intracellular amount and distribution of HBV RNAs (Fig. 4E). These results indicate that RAN’s activity is required for regulating the nuclear export of HBV RNAs.

Next, we examined whether RAN can form a nuclear export complex with CRM1 and RANBP3. In the presence of HBV pgRNA, we found that RANBP3 knockdown substantially reduced the interaction between RAN and CRM1, whereas both RAN T24N and RAN Q69L mutants completely abolished CRM1 binding (Fig. 4F and G). Consistently, the interaction of RAN T24N and RAN Q69L to CRM1 was much weaker than that of RAN WT in the control group (Fig. 4F and G). Together, these findings demonstrate that only active RAN can interact with CRM1-RANBP3 to mediate HBV RNA nuclear export.

### RANBP3 is required for CRM1-mediated HBV RNA nuclear export

**Fig 5:**
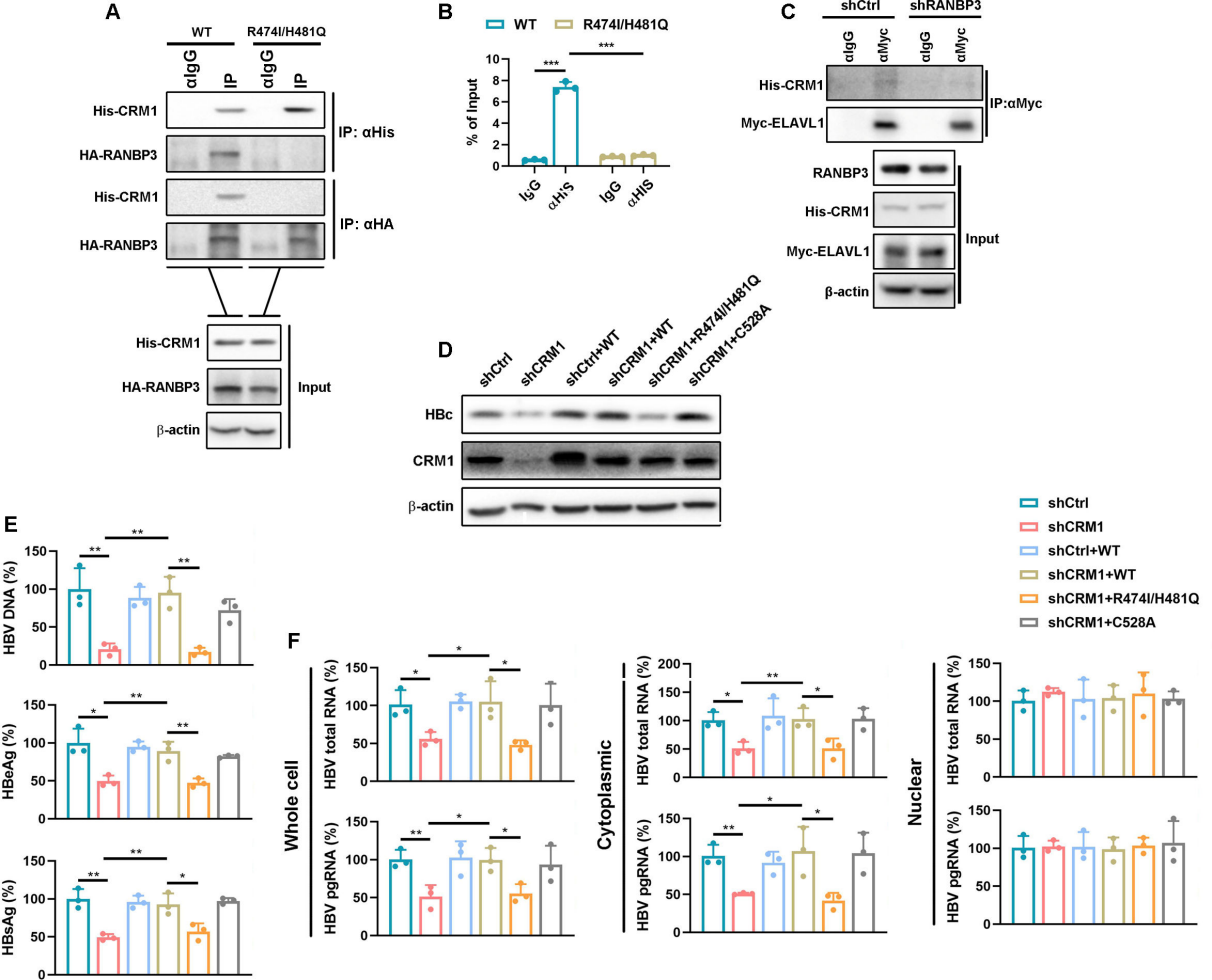
RANBP3 as a cofactor of CRM1 to promote HBV RNA export. (**A**) Co-transfect either His-CRM1 WT or His-CRM1 R474I/H481Q with HA-RANBP3 into Huh7 cells and incubate for 48 h. Co-IP assay was employed to detect the interaction between CRM1 and RANBP3. (**B**) Co-transfect His-CRM1 WT or His-CRM1 R474I/H481Q with pCDNA3.1-pgRNA into Huh7 cells for 48 h. RIP assay was used to determine the interaction between CRM1 and pgRNA. (**C**) Huh7 cells were transfected with RANBP3 target shRNA for 24 h. The cells were co-transfected with Myc-ELAVL1 and His-CRM1 plasmids. IP assay was used to detect the interaction between CRM1 and ELAVL1. (**D–F**) CRM1 stable knockdown HepG2-NTCP cells were transduced lentivirus-CRM1 WT, lentivirus-CRM1 R474I/H481Q, or lentivirus-CRM1 C528A. Then, the cells were infected with HBV at an MOI of 200 and maintained with DMEM containing 2.5% DMSO for 5 days. (**D**) WB was used to detect the protein level of HBc and CRM1. (**E**) The HBV DNA levels in the supernatant were detected by qPCR, and the secreted HBeAg and HBsAg levels in the supernatant were detected by ELISA. (**F**) HBV RNAs in different fractions were measured by qPCR. Data were shown as mean ± SD.*: *P* < 0.05, **: *P* < 0.01, and ***: *P* < 0.001.

To confirm the indispensable role of RANBP3 in CRM1-mediated nuclear export of HBV RNAs, we mutated two RANBP3-binding sites in CRM1, R474 and H481 (23). Using co-immunoprecipitation (Co-IP) assays, we verified that the CRM1 R474I/H481Q mutant completely lost its ability to bind RANBP3 compared with CRM1 WT (Fig. 5A). Consistently, the RIP assay demonstrated that CRM1 R474I/H481Q was unable to interact with HBV pgRNA (Fig. 5B). Moreover, IP assays demonstrated that RANBP3 knockdown reduced the interaction between ELAVL1 and CRM1 (Fig. 5C). Collectively, these findings demonstrate that RANBP3 is essential for bridging CRM1 to the ELAVL1-HBV RNA complex. Disruption of the CRM1-RANBP3 interaction abolishes CRM1 association with HBV pgRNA and weakens its interaction with ELAVL1, thereby impairing formation of an export-competent complex.

We next evaluated the effect of CRM1 R474I/H481Q on HBV RNA nuclear export. CRM1 C528A, which retains nuclear export function, was used as a negative control (24, 25). Unlike CRM1 WT, complementation with CRM1 R474I/H481Q (Fig. 5D) failed to recure HBV replication, as demonstrated by secreted HBeAg, HBsAg, and HBV DNA (Fig. 5E), and failed to rescue the nuclear export of HBV RNAs (Fig. 5F). Together, these data establish RANBP3 as a critical scaffold required for CRM1-mediated nuclear export of HBV RNAs.

### RAN is a key component essential for assembling the CRM1-HBV RNA nuclear export complex

**Fig 6:**
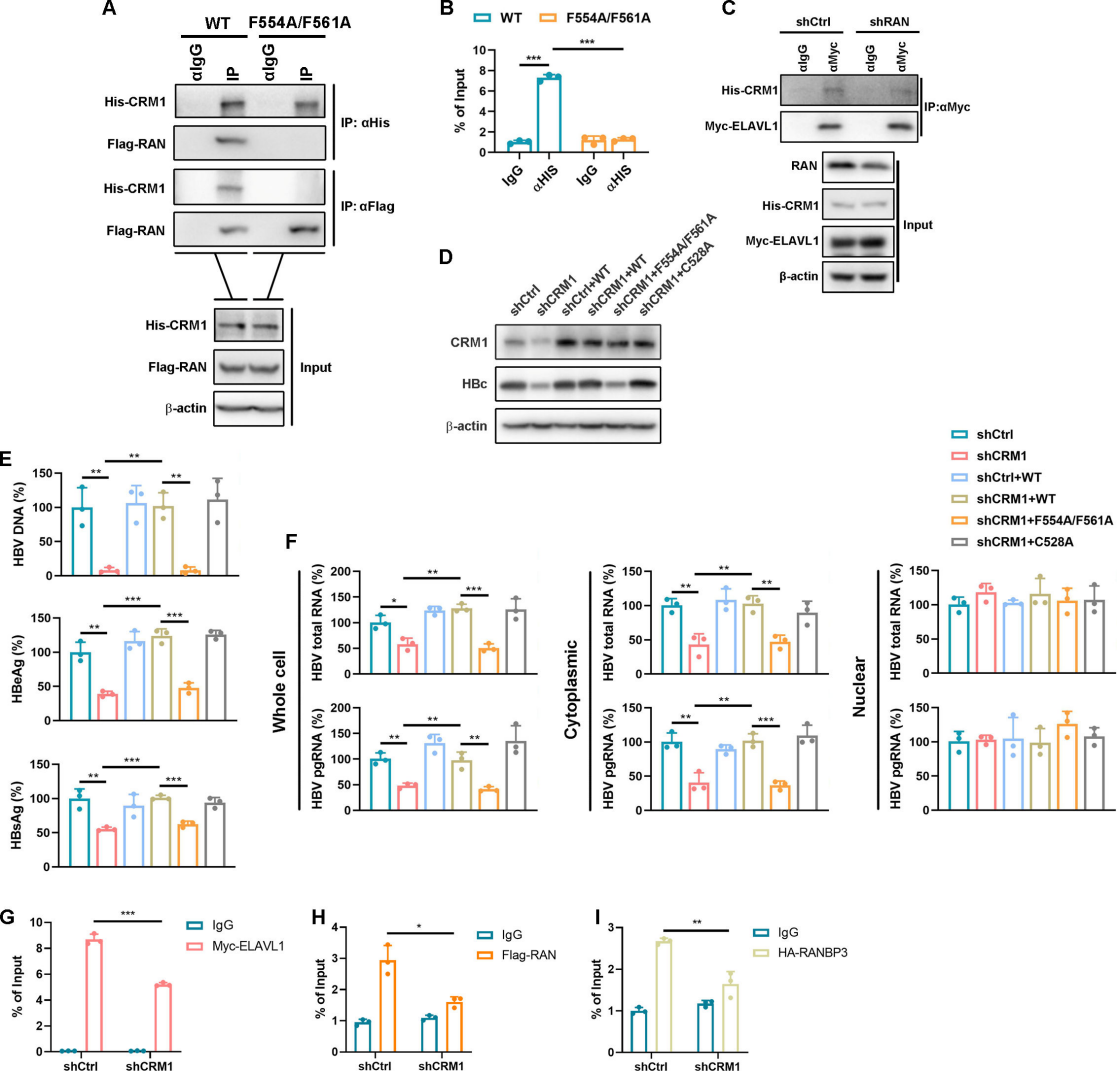
RAN is essential in the assembly of the CRM1 nuclear export complex. (**A**) Co-transfect either His-CRM1 WT or His-CRM1 F554A/F561A with FLAG-RAN into Huh7 cells and incubate for 48 h. Co-IP assay was employed to detect the interaction between CRM1 and RAN. (**B**) Co-transfect His-CRM1 WT or His-CRM1 F554A/F561A with pCDNA3.1-pgRNA into Huh7 cells for 48 h. RIP assay was used to determine the interaction between CRM1 and pgRNA. (**C**) Huh7 cells were transfected with RAN target shRNA for 24 h. The cells were co-transfected with Myc-ELAVL1 and His-CRM1 plasmids. IP assay was used to detect the interaction between CRM1 and ELAVL1. (**D–F**) CRM1 stable knockdown HepG2-NTCP cells were transduced with lentivirus-CRM1 WT, lentivirus-CRM1 F554A/F561A, or lentivirus-CRM1 C528A. Then, the cells were infected with HBV at an MOI of 200 and maintained with DMEM containing 2.5% DMSO for 5 days. (**D**) WB was used to detect the protein levels of HBc and CRM1. (**E**) The HBV DNA levels in the supernatant were detected by qPCR, and the secreted HBeAg and HBsAg levels in the supernatant were detected by ELISA. (**F**) HBV RNAs in different fractions were measured by qPCR. (**G–I**) Huh7 cells were transfected with CRM1 target shRNA for 24 h. The cells were co-transfected with Myc-ELAVL1, Flag-RAN, or HA-RANBP3 plasmids with pCDNA3.1-pgRNA plasmid. RIP assays were used to detect the interaction between ELAVL1-pgRNA, RAN-pgRNA, and RANBP3-pgRNA. Data were shown as mean ± SD.*: *P* < 0.05, **: *P* < 0.01, and ***: *P* < 0.001.

Since RAN loading is essential for CRM1-mediated nuclear export, we next sought to confirm the role of the RAN-CRM1 complex in HBV RNA export. We introduced mutations at residues F554 and F561 of CRM1, which are required for RAN binding, thereby disrupting formation of the CRM1 nuclear export complex (26). Co-IP assays confirmed that the CRM1 F554A/F561A mutant completely lost its interaction with RAN (Fig. 6A). Consistently, RIP assays showed that CRM1 F554A/F561A was unable to bind HBV pgRNA (Fig. 6B). Moreover, RAN knockdown reduced the interaction between ELAVL1 and CRM1 (Fig. 6C). Collectively, these findings demonstrate that RAN loading is essential for formation of a functional CRM1 export complex and for the stable association of CRM1 with ELAVL1-bound HBV RNAs, thereby supporting a critical role for the RAN-CRM1 axis in HBV RNA nuclear export.

To evaluate the functional impact of these mutations on HBV RNA export, we performed CRM1 complementation in the CRM1-stable knockdown cell line (Fig. 6D). The CRM1 F554A/F561A mutant failed to rescue HBV RNA export, as indicated by reduced secretion of HBeAg, HBsAg, and HBV DNA (Fig. 6E) and decreased cytoplasmic HBV RNAs (Fig. 6F). To further assess whether CRM1 functions as the central mediator linking RAN and RANBP3 to HBV RNAs, we performed RIP assays following CRM1 knockdown. Depletion of CRM1 diminished the association of ELAVL1, RAN, and RANBP3 with pgRNA, supporting the conclusion that CRM1 serves as a core scaffold bridging these factors to HBV RNAs (Fig. 6G through I). These results demonstrate that RAN binding is essential for CRM1 complex formation and for CRM1-mediated nuclear export of HBV RNAs.

**Fig 7:**
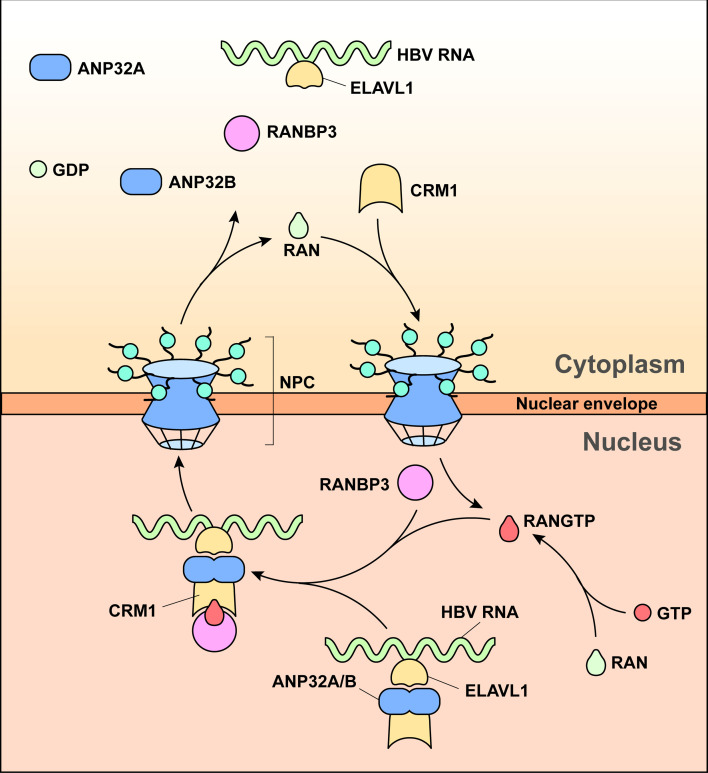
Graphical summaries. ELAVL1 recognizes the HBV RNAs AUUUA element and promotes their nuclear export via the CRM1 pathway. The addition of RANBP3 stabilizes the ELAVL1-HBV RNA-CRM1 nuclear export complex and recruits RAN-GTP to provide directionality and energy for the nuclear export of HBV RNAs.

Collectively, HBV relies on CRM1-dependent nuclear export of its viral RNAs, a process facilitated by ELAVL1 binding to AUUUA motifs on HBV transcripts. RANBP3 recruits RAN-GTP, enabling formation of a stable export-competent quaternary complex composed of CRM1, HBV RNAs, RANBP3, and RAN-GTP. This defines a precise regulatory axis in HBV RNA trafficking and positions RANBP3 and RAN as potential antiviral intervention points (Fig. 7).

## DISCUSSION

HBV replication and transcription occur in the nucleus, while translation takes place in the cytoplasm; therefore, the virus relies heavily on the host nuclear transport machinery. In our previous work, we demonstrated that HBV utilizes the CRM1 pathway to export its RNAs. Through pgRNA pulldown combined with LC-MS/MS, we identified ELAVL1 and CRM1 as key candidate factors. Specifically, ELAVL1 binds to AUUUA elements within HBV RNAs, stabilizing them, and its knockdown leads to a substantial reduction in the nuclear export of HBV RNAs (12). To further elucidate the molecular mechanisms governing HBV RNA export, in this study, we first examined the role of RANBP3 in the HBV life cycle. RANBP3 knockdown markedly reduced HBV antigen secretion and HBV DNA production in supernatants. Cytoplasmic-nuclear fractionation and RNAscope assays revealed a significant impairment in HBV RNA nuclear export. A similar phenotype was observed in mouse models; however, unlike the cell-based system, Ranbp3 knockdown also led to a marked decrease in nuclear HBV RNAs. This discrepancy may reflect differences between simplified *in vitro* models and the complex immune and physiological environments *in vivo*, and it warrants further investigation. The liver injury observed indicates RANBP3’s important role to maintain liver function. RAN knockdown likewise inhibited HBV RNA export and consequently suppressed HBV replication. Co-immunoprecipitation and CRM1 binding-site mutation experiments confirmed the interactions among CRM1, RANBP3, and RAN. Together, these findings indicate that RANBP3 and RAN function as essential cofactors in CRM1-mediated nuclear export of HBV RNAs. This study provides a more comprehensive and mechanistic understanding of HBV RNA nuclear export, showing that RANBP3 stabilizes the ELAVL1-HBV RNA-CRM1 export complex, while RAN-GTP supplies the directionality and energy required for efficient CRM1-dependent nuclear export.

Although our data indicate that HBV infection enhances RAN-GTP levels, the upstream regulatory mechanism remains to be fully defined. RCC1, the principal guanine nucleotide exchange factor (GEF) for RAN, catalyzes GDP-GTP exchange and establishes the nuclear RAN-GTP pool required for CRM1-mediated export (27, 28). HBV may modulate RAN activity by affecting GEF protein expression, stability, subcellular localization, or post-translational modifications or by altering the balance between GEF and GAP activities that collectively shape the RAN-GTP gradient. Further investigation will be necessary to elucidate how HBV reprograms this regulatory network to optimize viral RNA nuclear export.

Given the central role of RAN in regulating nucleocytoplasmic transport, it is important to consider that its perturbation may exert broader, indirect effects on host cell physiology that could influence HBV RNA export (29). In addition to its direct involvement in CRM1-dependent export, altered RAN activity may impact the nuclear import and export of host mRNAs and proteins, including key components of the RNA processing and export machinery, as well as transcriptional regulators that govern viral gene expression (30). Such global changes in nucleocytoplasmic trafficking could secondarily affect the intracellular distribution of HBV RNAs. Therefore, while our data support a model in which the RAN-CRM1 axis contributes to HBV RNA nuclear export, we cannot exclude the possibility that part of the observed phenotype arises from indirect effects mediated through broader disruption of host transport pathways.

CRM1 is a universal receptor for the nuclear export (31, 32), which mediates the export of approximately 200 proteins, several mRNAs, and small-nuclear RNAs to cytoplasm (27, 28). The activity and specificity of CRM1 are regulated by various cofactors, including RANBP3 and RAN (33–35). RANBP3 stabilizes the CRM1 nuclear export complex and increases nuclear export efficiency (33). Lindsay et al. showed that the human ortholog of Yrb2p (RANBP3) participates in the regulation of Crm1 nuclear export as a cofactor (34). They found that Yrb2p can directly bind to Crm1 and that knocking down Yrb2p disrupts Crm1-mediated nuclear export in yeast. The small GTPase RAN plays an essential role in regulating nuclear export. The alternating binding of GTP and GDP is pivotal for the directionality and energy of transport between the nucleus and the cytoplasm (36–38). RANBP3 recruits the GTP-bound form RAN to the CRM1 cargos to form an export complex (27, 39). Once the complex has passed through the nuclear pore into the cytoplasm, RAN-GTP is hydrolyzed to RAN-GDP, thereby dissociating the complex and releasing the cargo into the cytoplasm (27, 32).

It has been well documented that several viruses transport their proteins and transcripts through the RANBP3-RAN-CRM1 nuclear export pathway (40–42). For example, influenza A relies on the RANBP3/RAN/CRM1 complex to transport its viral ribonucleoprotein (vRNP) to the cytoplasm for assembly (43, 44). Knockdown of RANBP3 led to vRNP nuclear retention (45). During the early phase of human immunodeficiency virus type 1 (HIV 1) replication, viral Rev protein, which contains an NSL sequence, is capable of translocating to the nucleus (46, 47). There, Rev forms a dimer and recruits CRM1/RAN-GTP to mediate the nuclear export of unspliced and partially spliced HIV-1 transcripts to the cytoplasm (48).

Our findings highlight RANBP3 and RAN as previously unrecognized host dependency factors that are essential for HBV RNA nuclear export and viral propagation, offering new avenues for therapeutic intervention. Targeting the RANBP3-RAN-CRM1 axis could provide a strategy to selectively disrupt the nuclear export of HBV transcripts, thereby inhibiting viral antigen production and replication. Importantly, both RANBP3 and RAN function downstream of CRM1 to stabilize and energize the export complex, suggesting that partial inhibition of their activity may achieve antiviral effects while minimizing global disruption of CRM1-mediated export. Pharmacologic inhibitors of the RAN GTPase cycle or molecules that interfere with RANBP3-CRM1 or RAN-CRM1 interactions could, therefore, be developed as host-targeted antivirals with a high barrier to resistance. Given that existing HBV therapies do not eliminate cccDNA or suppress viral antigen production sufficiently, targeting this essential export machinery may complement current treatments and contribute to achieving a functional cure (8). Future efforts to identify specific inhibitors or degraders of RANBP3 or RAN and to evaluate their safety and antiviral efficacy *in vivo* could open a promising therapeutic direction for chronic HBV infection.

## MATERIALS AND METHODS

### Cell culture and transfection

For HepG2-NTCP cells and HepaAD38 cells, culture flasks were precoated with 50 μg/mL rat tail collagen I rat tail (Corning, USA) at room temperature for 1–2 h. Cells were then seeded into the coated flasks and cultured in Dulbecco’s modified Eagle medium (DMEM) containing 10% fetal bovine serum (FBS) and 1% penicillin-streptomycin (PS) at 37°C in a 5% CO₂ incubator. For Huh7 cells and HEK293T cells, cells were seeded into culture flasks and cultured in DMEM containing 10% FBS and 1% PS at 37°C in a 5% CO₂ incubator. We used Lipofectamine RNAiMAX (Invitrogen, USA) for siRNA transfection. The siRNA sequences are provided in Table 1.

**TABLE 1:** siRNA sequences

Oligo name	Sequence
siRANBP3-1	GACAGAGTTAAGCTGATAAAT
siRANBP3-2	AGAGACAGAGTGAAGTTAAT
siRAN-1	GCACAGTATGAGCACGACTTA
siRAN-2	GACCCTAACTTGGAATTTGTT

### Experimental animals

Male C57BL/6J mice (6–8 weeks old) from China Three Gorges University Laboratory Animal Center (Yichang, China) were randomly assigned to two groups: (i) the control group (AAV-HBV1.2 + AAV-shCtrl) and (ii) the knockdown group (AAV-HBV1.2 + AAV-shRanbp3). Mice received a single tail vein injection containing AAV-HBV1.2 (2.5 × 10¹¹ vg) combined with either AAV-shCtrl or AAV-shRanbp3 (5.0 × 10¹⁰ vg) in 200 μL phosphate-buffered saline (PBS). Serum and liver tissues were collected at designated time points for analysis.

### Western blotting

Proteins were extracted from cells or tissues using RIPA buffer (Beyotime, China) containing protease and phosphatase inhibitors. Protein concentrations were determined by a bicinchoninic acid (BCA) assay (P0012, Beyotime, China). Equal amounts of protein were separated by SDS-PAGE and transferred to polyvinylidene fluoride (PVDF) membranes (IPVH00010, Millipore, MA, USA). After blocking with 5% skim milk for 1 h, the membranes were probed with specific primary antibodies overnight at 4°C, followed by incubation with horseradish peroxidase (HRP)-conjugated secondary antibodies. The chemiluminescent signals were detected and quantified using a GeneGnome XRQ chemiluminescence imaging system (XRQ-NPC, GeneGnome, Hong Kong, China). The antibodies are listed in Table 2.

**TABLE 2:** Antibodies

Antibody	Company	Catalog no.	Dilution(s)
Rabbit anti RANBP3	Solarbio (Beijing, China)	K014662RR	WB: 1:1,000, IF: 1:100
Rabbit anti RAN	Proteintech (Wuhan, China)	10469-1-AP	WB: 1:1,000
Rabbit anti-HBs	NOVUS Biology (Littleton, CO, USA)	NB10062652	WB: 1:1,000, IHC: 1:200
Rabbit anti-HBc	Self-made	N/A^*a*^	WB: 1:1,000, IHC: 1:50
Rabbit anti-β-actin	ABclonal (Wuhan, China)	AC026	WB: 1:10,000, IF: 1:200
Mouse anti-Myc	BIOPRIMACY (Wuhan, China)	PMK112M	IP: 1:100
Mouse anti-His	BIOPRIMACY (Wuhan, China)	PMK0115	IP: 1:100
Anti-rabbit IgG, HRP-linked	Cell Signaling Technology (Danvers, MA, USA)	7074	WB: 1:10,000
Anti-mouse IgG, HRP-linked	Cell Signaling Technology (Danvers, MA, USA)	7076	WB: 1:10,000
Rabbit IgG	Proteintech (Wuhan, China)	B900610	IP: 1:200
Mouse IgG	Proteintech (Wuhan, China)	B900620	IP: 1:200

^*a*^ N/A, not applicable.

### ELISA

The serum and cell culture supernatants HBeAg and HBsAg were determined by enzyme-linked immunosorbent assay (ELISA) (Kehua Bio-Engineering Co., China). The absorbance was read at 450 nm using a microplate reader.

### RT-qPCR

Total RNA was extracted from samples using TRIzol reagent following the manufacturer’s protocol. cDNA was synthesized from 500 ng RNA using a PrimeScript RT kit (FSQ-301, TOYOBO, Japan). Quantitative PCR was performed with FastSmart Essential DNA Green Master Mix (06924204001, Roche, Germany) on LightCycler 480 II. Primers used for qPCR are listed in Table 3.

**TABLE 3:** Primer sequences for RT-qPCR, q-PCR, and PCR

Oligo name	Forward primer sequence	Reverse primer sequence
HBV pgRNA	CTGGGTGGGTGTTAATTTGG	TAAGCTGGAGGAGTGCGAAT
HBV total RNA	CCGTCTGTGCCTTCTCATCTGC	ACCAATTTATGCCTACAGCCTCC
RANBP3	TTGGAAAAAGTGGAAGTCATCA	CAAACAGCTTGCACTGCATC
RAN	AAGTTCAATGTATGGGACACAGC	TCAAACATTATGATGGCACACTG
β-actin	ATCGTGCGTGACATTAAGGAG	GGAAGGAAGGCTGGAAGAGT
Human snU2	ATGGATTTTTGGAGCAGGGAGA	GCACCGTTCCTGGAGGTACTG
Mouse snU1	GGAGATACCATGATCACGAAGG	AGTCGAGTTTCCCGCATTT
shRanbp3-1	ACCCCAAAGCTGAATGAAGCCAATC TCGAGATTGGCTTCATTCAGCTTTGG	AACCCAAAGCTGAATGAAGCCAATC TCGAGATTGGCTTCATTCAGCTTTGG
shRanbp3-2	ACCGAGAGACAGAGTGAAGTTAATCT CGAGATTAACTTCACTCTGTCTCTC	AACGAGAGACAGAGTGAAGTTAATC TCGAGATTAACTTCACTCTGTCTCTC
shRANBP3-1	CCGGGACAGAGTTAAGCTGATAAATCTC GAGATTTATCAGCTTAACTCTGTCTTTTTG	AATTCAAAAAGACAGAGTTAAGCTGA TAAATCTCGAGATTTATCAGCTTAACTCTGTC
shRANBP3-2	CCGGTGAATTGAAATGGCACATTAACTC GAGTTAATGTGCCATTTCAATTCATTTTTG	AATTCAAAAATGAATTGAAATGGCACATT AACTCGAGTTAATGTGCCATTTCAATTCA
shRAN-1	CCGGGCACAGTATGAGCACGACTTACTC GAGTAAGTCGTGCTCATACTGTGCTTTTTG	AATTCAAAAAGCACAGTATGAGCACGACT TACTCGAGTAAGTCGTGCTCATACTGTGC
shRAN-2	CCGCGACCCTAACTTGGAATTTGTTCTCGA GAACAAATTCCAAGTTAGGGTCTTTTTG	AATTCAAAAAGACCCTAACTTGGAATTTGTT CTCGAGAACAAATTCCAAGTTAGGGTC
HBV cccDNA	GCCTATTGATTGGAAAGTATGT	AGCTGAGGCGGTATCTA

### Virus production and infection

Lentiviral particles were produced by co-transfecting HEK293T cells with pLKO.1-shRNA plasmids, the packaging plasmid (psPAX2), and the envelope plasmid (pMD2.G) using the PEI MAX 40K Reagent (24765-1, Polysciences, USA). The viral supernatant was harvested at 48 h and 72 h post-transfection, concentrated by ultracentrifugation, and titrated on HEK293T cells. For infection, target cells were transduced with the viral stock in the presence of 8 μg/mL polybrene.

Recombinant AAV was produced by co-transfecting HEK293T cells with the pX552-shRNA, pAAV2/8RC, and pHelper plasmids at a 1:1:1 molar ratio using PEI MAX 40K Reagent. The viral supernatant was collected 72 h post-transfection, filtered through a 0.22-μm membrane, and concentrated with the Cytiva protein concentration unit (28932363, Cytiva, USA). The genomic titer (vg/mL) was determined by qPCR.

HBV was harvested from the culture supernatant of HepAD38 cells. The supernatant was concentrated using a 100-kDa centrifugal filter (UFC910096, Millipore, USA), and the viral genomic titer was quantified using a commercial HBV DNA diagnostic kit (Sansure Biotech, China).

HepG2-NTCP cells were infected with HBV at a multiplicity of infection (MOI) of 200. The infection was carried out in the culture medium supplemented with 4% PEG 8000 and 2.5% DMSO for 24 h. Following incubation, the cell monolayer was rigorously washed five times with PBS to remove unbound virions. The cells were then maintained in fresh complete medium containing 2.5% DMSO. The culture supernatant and cells were harvested at the indicated time points post-infection for subsequent analysis. The primer sequences are provided in Table 3.

### Co-immunoprecipitation

Cells were lysed in NP-40 lysis buffer (N8030, Solarbio, China) containing protease inhibitors. Cleared lysates were incubated overnight at 4°C with antibody or control IgG. Protein A/G agarose beads (SA032025, Smart-Lifesciences, Changzhou, China) were then added for 4 h to capture the immune complexes. The beads were washed extensively with lysis buffer, and the bound proteins were eluted by boiling in SDS loading buffer. The input lysate and co-immunoprecipitated samples were analyzed by Western blotting (WB) to detect protein interactions. The antibodies are listed in Table 2.

### RNA immunoprecipitation

Approximately 1 × 10⁷ cells were collected per immunoprecipitation reaction. Cells were pelleted, washed with ice-cold PBS, and lysed in polysome lysis buffer (100 mM KCl, 5 mM MgCl_2_, 10 mM HEPES [pH 7.0], 0.5% NP-40, 1 mM DTT, 400 μM VRC, and RNase-DNase-free H_2_O) supplemented with RNase and protease inhibitors. The lysate was incubated on ice, clarified by centrifugation at 15,000 × *g* for 15 min, and the supernatant was collected as the input lysate. Protein A-/G-coated agarose beads were pre-blocked with 5% BSA in NT2 buffer (50 mM Tris HCl [pH 7.4], 150 mM NaCl, 1 mM MgCl_2_, 0.05% NP-40, and RNase-DNase-free H_2_O) buffer for 1 h at 4°C. The beads were then incubated with the target-specific antibody or control IgG for 4 h at 4°C with gentle rotation to form antibody-coated beads. For each RIP reaction, 100 μL of the cleared lysate was incubated with the antibody-coated beads for 4 h at 4°C. The beads were then pelleted and washed four times with 1 mL of ice-cold NT2 buffer. Co-precipitated RNAs were finally eluted and purified using TRIzol reagent, and the enrichment of specific RNAs was analyzed by RT-qPCR. The primer sequences are provided in Table 3. The antibodies are listed in Table 2.

### RNA pulldown

The linearized pCDNA3.1-pgRNA plasmid was used as a template to synthesize desthiobiotinylated RNA probes in an *in vitro* transcription kit (MSC11610, CELLSCRIPT, USA). The reaction products were treated with DNase I to remove the DNA template, followed by purification via an RNA purification kit (5401050, SIMGEN, China). Both wild-type pgRNA and mutant control probes were prepared simultaneously. Streptavidin-coated magnetic beads were activated and washed sequentially with an alkaline solution (0.1 M NaOH and 0.05 M NaCl) and 0.1 M NaCl. The purified, desthiobiotinylated RNA probes were diluted in 2× Washing & Binding (W&B) buffer (5 mM Tris-HCl, pH 7.4) and incubated with the prepared beads for 1 h at room temperature to allow for immobilization. The RNA-bound beads were then washed twice with 1× W&B buffer containing 1 M NaCl to remove unbound RNA. Pre-cleared cell lysates were incubated with the RNA-immobilized beads for 3 h at 4°C with gentle rotation. Subsequently, the beads were collected and washed stringently with W&B buffer. Specifically bound proteins were competitively eluted using 2 mM biotin in PBS for 3 h at room temperature. The resulting eluates were analyzed by LC-MS/MS assays.

### LC-MS/MS

Proteins captured by the RNA pulldown assay (as described in “RNA pulldown,” above) were subjected to on-bead digestion in urea buffer. The resulting peptides were desalted using C18 StageTips (Thermo Fisher Scientific) and analyzed by an Orbitrap Exploris 480 mass spectrometer equipped with a FAIMS Pro interface.

### RNAscope

The subcellular distribution and expression levels of HBV RNAs in cultured cells and formalin-fixed, paraffin-embedded (FFPE) liver tissue sections were detected using the RNAscope platform. Sample pretreatment and hybridization were performed using the RNAscope Sample Preparation Kit and the RNAscope Multiplex Fluorescent Reagent Kit, respectively (322381, Advanced Cell Diagnostics, USA), following the manufacturer’s instructions. Fluorescent signals were captured using confocal laser scanning microscopy. The cell and tissue were imaged using a Leica TCS SP8 system (software: LAS X Version 3.4.2.18368). The following filter sets were applied for all acquisitions: DAPI/Hoechst channel (excitation 405 nm; emission 417–480 nm) for nuclear counterstaining and the probe dye 570 (excitation 561–587 nm; emission 570–690 nm) for the specific detection of the HBV RNA probe signal.

### Nuclear and cytoplasmic RNA extraction

Nuclear and cytoplasmic fractions were isolated from adherent cells and mouse liver tissues using a commercial extraction reagent kit (P00028, Beyotime, China), following the manufacturer’s protocol.

For adherent cells, samples were harvested by trypsinization, collected by centrifugation at 500 × *g* for 5 min, and washed twice with ice-cold PBS. The cell pellet was processed immediately for fractionation. For mouse liver tissue, approximately 60 mg of the sample was dissected into small pieces, washed with ice-cold PBS, and homogenized in the provided cytoplasmic extraction reagent using a mechanical homogenizer. The subsequent fractionation steps for both cell and tissue samples were performed identically according to the kit instructions.

### GTPase activity assay

GTPase activity was measured using a commercial GTPase Activity Assay Kit (P2435, Beyotime, China) according to the manufacturer’s instructions. Briefly, whole-cell proteins were incubated with the provided GTP substrate in assay buffer at 37°C for 1–2 h. The enzymatic reaction produces GDP, which is subsequently quantified through a coupled enzyme reaction that generates a colorimetric (absorbance at 650 nm) or fluorometric signal. The signal intensity, measured using a microplate reader, is directly proportional to the GTPase activity in the sample.

### RAN-GTP pulldown

Cellular RAN-GTP levels were assessed using the RAN pulldown Activation Assay Kit (26915, Wuhan National Bio-industry, China) according to the manufacturer’s protocol. In principle, a mouse monoclonal antibody that specifically recognizes the active conformation of RAN can selectively bind to the GTP-bound, active form of RAN (RAN-GTP) in cell lysates. The antigen-antibody complexes are then captured using Protein A/G beads. Subsequently, the precipitated active RAN is detected by immunoblotting using a rabbit polyclonal antibody that recognizes total RAN.

### Myrcludex B treatment

HepaG2-NTCP cells were maintained with DMEM supplemented with 2.5% dimethyl sulfoxide (DMSO) for 2 days. The cells were then infected with HBV and supplemented with 500 nM of Myrcludex B (HY-P3465, MedChemExpress, China) for 24 h. Subsequently, the cells were washed with PBS three times.

### qPCR for cccDNA quantification

Genomic DNA was extracted from cells, and 500 ng of total DNA was treated with 5 units of T5 exonuclease (M0663L, New England Biolabs, USA) in a 10 μL reaction volume at 37°C for 30 min to selectively digest non-cccDNA species (including rcDNA and linear DNA). The enzyme was subsequently heat-inactivated at 95°C for 5 min, and the treated DNA was diluted tenfold with distilled water. qPCR was performed on a LightCycler480 II instrument using FastStart Essential DNA Green Master (06924204001, Roche, Germany). A standard curve was generated using 1× HBV DNA derived from the EcoRI-digested pSM2 plasmid. The primer sequences used for cccDNA detection are listed in Table 3.

## Data Availability

The raw data of LC-MS/MS are available on ProteomeXchange Consortium (reference number: 1-20230724-064428-2501911; project accession ID: PXD044015) and are publicly accessible at https://proteomecentral.proteomexchange.org.
